# Agreement between 4D transesophageal echocardiography and multi-detector computed tomography in measuring aortic root dimensions and coronary ostia heights

**DOI:** 10.1007/s10554-023-02873-3

**Published:** 2023-06-03

**Authors:** Mohamed Hassan, Mostafa M. Abdrabou, Wasseem Amin Wahba, Amir Anwar Samaan, Yasser Baghdady, Ahmed A. Elamragy

**Affiliations:** 1 Department of Cardiology, Faculty of Medicine, Cairo University, Cairo, Egypt; 2 Department of Cardiology, Al Nas Hospital, Qalyubia, Egypt

**Keywords:** Aortic annulus, Coronary ostial height, 4D TEE, MDCT

## Abstract

Multi-detector computed tomography (MDCT) is the gold standard non-invasive tool for evaluating aortic root dimensions. We assessed the agreement between 4D TEE and MDCT-derived aortic valve annular dimensions, coronary ostia height, and minor dimensions of sinuses of Valsalva (SoV) and sinotubular junction (STJ). In this prospective analytical study, we measured the annular area, annular perimeter, area-derived diameter, area-derived perimeter, left and right coronary ostial heights, and minor diameters of the SoV and the STJ using ECG-gated MDCT and 4D TEE. TEE measurements were calculated semi-automatically by the eSie valve software. We enrolled 43 adult patients (27 males, median age: 46 years). We found strong correlations and good agreement between the two modalities in annular dimensions (area, perimeter, area-derived diameter, and perimeter-derived diameter), left coronary ostial height, minimum STJ diameter, and minimum SoV diameters. Moderate correlations, and agreement, with relatively large differences between the 95% LOA, were demonstrated for the right coronary artery ostial height. 4D TEE correlates well with MDCT in measuring aortic annular dimensions, coronary ostial height, SoV minor diameter, and sinotubular junction minor diameter. Whether this can affect clinical outcomes is unknown. It could replace MDCT if the latter is unavailable or contraindicated.

## Introduction

Because of the development in transcatheter aortic valve replacement procedures and the refinement of aortic root surgeries, non-invasive aortic root evaluation has become an increasingly essential part of the clinical practice of cardiologists, cardiac surgeons, and radiologists. The availability of a correct and reliable non-invasive technology substantially impacts clinical decisions and outcomes in aortic root pathologies and aortic valve illnesses. Multidetector computed tomography (MDCT) is currently the method of choice for measuring aortic root diameters [1]. Although MDCT has excellent temporal and spatial resolutions, it carries the risk of radiation and iodinated contrast [2]. It also has special implications in renal disease and iodinated compound allergy. These drawbacks, combined with the improvements in transesophageal echocardiography (TEE) technology, led to the increasing use of four-dimensional TEE (4D-TEE) in measuring the annulus size and aortic root dimensions. In this study, we investigated the agreement between semi-automated 4D-TEE and MDCT in measuring the aortic root dimensions and the coronary ostial heights.

## Materials and methods

This was a single-center, cross-sectional observational study done in Al Nas Hospital between June 2020 and February 2022. We included 43 patients who already did an MDCT exam, either as a workup for transcatheter aortic valve replacement (TAVR), as a pre-operative coronary evaluation before non-coronary cardiac surgery, or as a part of an evaluation of the cardiac disease. We did 4D-TEE exam for all patients (either before TAVR or intraoperatively). We excluded patients under 18 years of age, those with atrial fibrillation, bicuspid aortic valves, contraindications to CT angiography (renal impairment history of allergy to ionic contrast, pregnancy), or contraindications to TEE (esophageal stricture or recent hematemesis).

### 4D-TEE

Electrocardiogram (ECG)-gated 2D and 4D TEE were performed according to the guidelines [3] using a commercially available TEE transducer (Z6M transducer; Siemens AG, Munich, Germany). The best mid-esophageal view was used to acquire the 4D TEE images [4].

We analyzed the data sets using the eSie valve software on the same machine (Siemens Acuson SC2000 prime ultrasound system; Siemens AG, Munich, Germany). 3D data sets were manually adjusted to include the whole aortic root, and the maximum valve opening phase identified the systole.

**Fig. 1:**
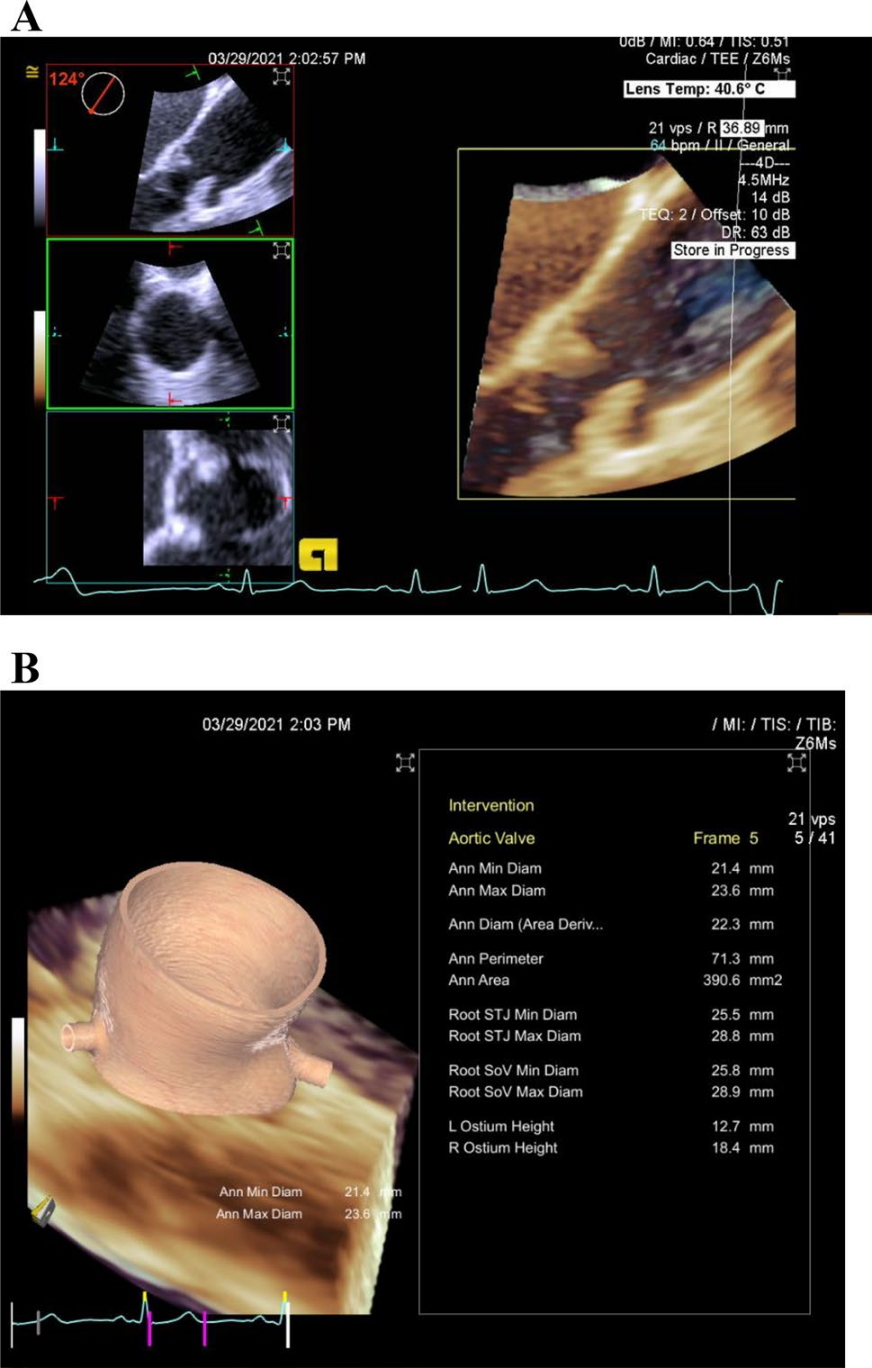
4D TEE Aortic root measurements. **A** Acquired 3D data set with selected systolic phase for analysis and **B** software generated report with 3D reconstructed model

 The eSie valve software determines the aortic root measurements automatically. Based on the obtained 3D dataset, the software generates a virtual model by recognizing anatomical landmarks such as valve commissures, leaflet hinges, and coronary ostia. This technology is based on a vast image database used to train artificial intelligence algorithms for image identification, allowing for robust virtual 3D modeling. Then, using automated tracking techniques, these algorithms fit the patient-specific data to a valve surface model that connects the identified landmarks [4] (Fig. 1).

Then, the aortic annular area, aortic annular perimeter, area-derived annular diameter, aortic annulus major and minor diameters, sino-tubular junction (STJ) maximum and minimum dimensions, SoV maximum and minimum diameters, and coronary ostial heights are automatically exported to a software-generated report and an excel sheet (Fig. 1).

### MDCT

All examinations were carried out on a Siemens SOMATOM drive 128-slice CT scanner (Siemens AG, Munich, Germany) with the following standard technical parameters: gantry rotation time—0.33 ms; axial coverage—0.5 mm [128 × 0.6 mm]; tube voltage [weight-adjusted]—0–120 kV; milliampere intensity with Care Dose 4D modulation; and temporal resolution—70 ms. The ECG gated the images, which were then acquired during a breath hold. Contrast enhancement was achieved using 60–80 mL of iopromide (Ultravist 370 mg/mL). We used a bolus tracking method in the ascending aorta for optimal synchronization. An additional dose of oral propranolol (20–40 mg) was administered to lower the heart rates that were > 70 bpm at the time of the study, and the examination was not continued at such high heart rates. The thickness of reconstructed images was 0.5 mm [5].

**Fig. 2:**
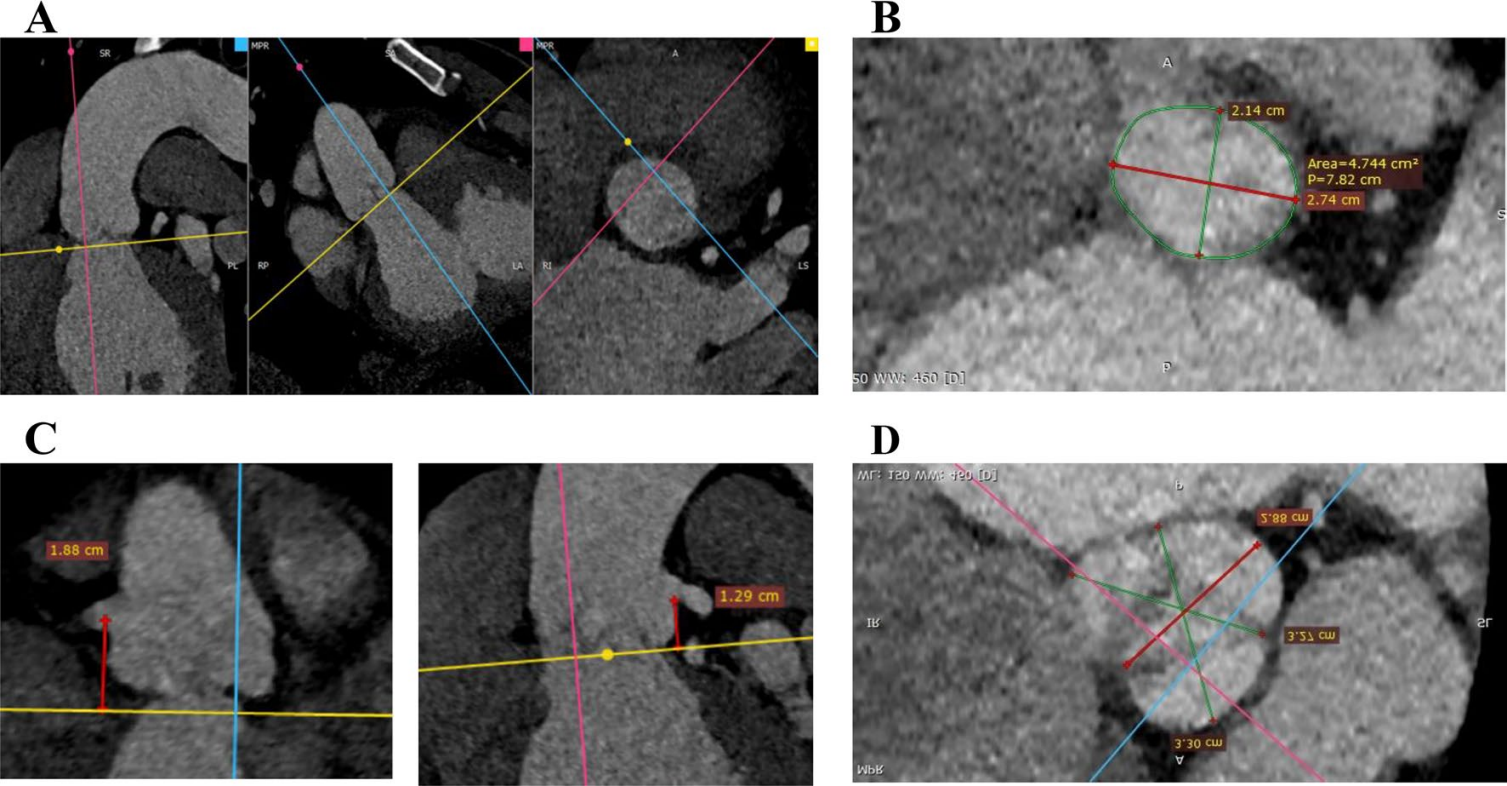
MDCT derived aortic root measurements. **A** MPR image showing the leveling of the annulus. **B** Tracing of the aortic annulus. **C** Measurement of the right coronary height and the left coronary height. **D** SoV measurements

Aortic annulus measurements were done in multiplanar reconstruction images (MPR) using a dedicated software during the best systolic phase (35–45%), which was chosen after examining the acquired phases in the axial cuts [5–7]. The aortic annulus is the virtual ring located just below the basal attachments of all three valvular cusps. MPR images were oriented manually to show the aortic annulus at basal attachment points. Two orthogonal planes, bisecting the aortic valve in sagittal and coronal planes, were manually set. The third orthogonal plane (double-oblique transverse view) was set to bisect the aortic annulus at the most caudal attachment points of all three native cusps, orientating/positioning the virtual basal ring optimally [5]. The aortic annulus’s outer border was manually traced. A specific software calculated the annular area, perimeter, area-derived diameter, and minimum and maximum diameters based on this tracing [5]. The heights of the left and right coronary arteries (LCA and RCA, respectively) were measured in relation to the previously defined annular plane. The MPR was also used to identify the SoV and STJ planes, and the maximum and minimum diameters of both were measured (Fig. 2).

### Aortic annular measurements, definitions, and geometric analysis

Measurements derived from 4D-TEE and MDCT data sets were as follows: Aortic annular area (mm^2^), Annular perimeter (mm). Area-derived annular diameter and perimeter-derived annular diameter were calculated as follows [8]:

 

$${\text{Area-derived annular diameter}} = 2 \times \sqrt {Area/\pi }$$

  

 

$${\text{Perimeter-derived annular diameter}} = \frac{{Perimeter}}{\pi }$$

  

An experienced echocardiographer performed the TEE and acquired the 4D TEE data, while an experienced radiologist did the MDCT analysis: both were blinded to the measurements from the other modality.

### Statistical analysis

Data analysis was applied using Jamovi software version 2.2.5. The normal distribution of data was checked using the Shapiro–Wilk test. Descriptive data were expressed as median and interquartile range (IQR) if the data were skewed and mean and standard deviation (SD) if normally distributed. Categorical data were expressed as numbers and percentages. The correlation between TEE and MDCT-derived dimensions was assessed using the Pearson correlation test and calculating the concordance correlation coefficient. Bland–Altman analysis and plot were used to evaluate the agreement between TEE and MDCT in measuring different aortic parameters [9].

## Results

Among 218 adult patients (183 undergoing open heart surgery and 35 undergoing elective TEE on an outpatient basis), 43 met the inclusion criteria and completed the study protocol.

The median age of the patients was 46 years. The most common indication for an MDCT scan was a pre-operative evaluation of coronary arteries before non-coronary cardiac surgery (n = 32). Eleven patients had severe aortic stenosis (AS): 3 degenerative and 8 rheumatic (Table 1).

**Table 1:** Characteristics of the study population

Variable	N (%)
Demographics	
Age, years^a^	46 (18–72)
Male gender	27 (62.7%)
Risk factors and comorbidities	
Systemic hypertension	16 (37.1%)
Type 2 diabetes mellitus	9 (20.9%)
Serum creatinine, mg/dl^b^	0.93 ± 0.15
Body weight, kg^b^	79.2 ± 17.4
Body mass index, kg/m^2 b^	27.4 ± 7.2
Indications for CT and TEE	
Pre-operative coronary angiography	32 (74.4%)
Severe aortic stenosis	11 (25.5%)
Other valvular disease	14 (32.5%)
Coronary bypass surgery	7 (16.2%)
Cardiac CT for congenital heart disease	13 (30.2%)

*CT* computed tomography, *TEE* transesophageal echocardiography

^a^Median, interquartile range

^b^Mean ± standard deviation

**Table 2:** Comparison of 4D TEE and MDCT-derived aortic root dimensions

	4D-TEE	MDCT derived	Difference (%)
Mean annular area (mm^2^)	466.2	494.8	− 28.6 (5.7)
Mean annular perimeter (mm)	77.2	80.4	− 3.2 (4)
Area-derived annular diameter (mm)	24.0	24.8	− 0.8 (3.2)
Perimeter-derived annular diameter (mm)	24.5	25.5	− 1.0 (3.5)
Left coronary height (mm)	14.0	14.2	− 0.2 (1.5)
Right coronary height (mm)	16.6	16.1	+ 0.5 (3.1)
STJ maximum diameter (mm)	29.3	30.5	− 1.2 (3.9)
STJ minimum diameter (mm)	26.6	28.1	− 1.5 (5.3)
SoV maximum diameter	31.2	33.2	− 2.0 (6)
SoV minimum diameter (mm)	27.6	29.5	− 1.9 (6.4)

*4D TEE *four-dimensional transesophageal echocardiography, *MDCT* multidetector computed tomography, *STJ* sinotubular junction, *SoV* sinuses of valsalva

The annular area, annular perimeter, area-derived annular diameter, perimeter-derived annular diameter, STJ, and SoV dimensions derived from 4D TEE were smaller than those of the MDCT. These differences were statistically significant, except for maximum STJ junction and maximum SoV dimensions. However, the 4D TEE-derived RCA average ostial height was greater than that of the MDCT, while there was minimal difference in the LCA ostial height (− 0.2 mm). But these differences did not reach statistical significance (Table 2).

### Correlations and agreement

**Fig. 3:**
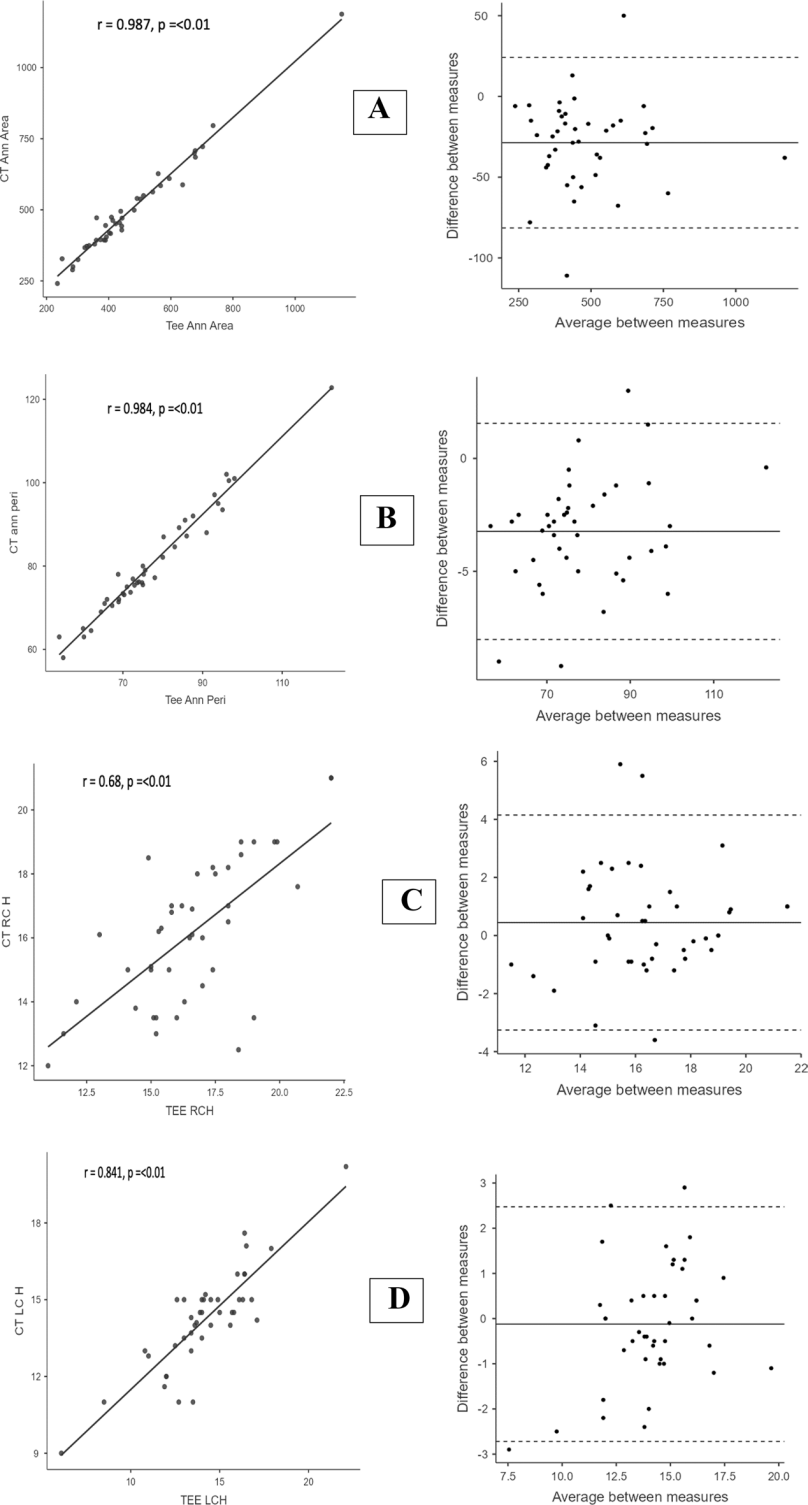
**A** Scatter plot (left) and Bland–Altman plot (right) of annular area measurements. **B** Scatter plot (left) and Bland–Altman plot (right) of annular perimeter measurements. **C** Scatter plot (left) and Bland–Altman plot (right) of right coronary artery ostial height **D** Scatter plot (left) and Bland–Altman plot (right) of left coronary artery height. *CT* computed tomography, *Ann* annulus, *TEE* trans-esophageal echocardiography, *Peri* perimeter, *RCH* right coronary height, *LCH* left coronary height

The 4D-TEE and MDCT-derived annular area measurements had very strong and significant positive linear correlation and showed good agreement (r = 0.987, p ≤ 0.01, CCC = 0.972, and ICCC = 0.974). The 95% limits of agreement (LOA) for Bland–Altman analysis were − 81.4 mm^2^ and 24.2 mm^2^, with a bias of − 28.6 mm^2^. Most data points were within the 95% LOA. The 4D-TEE and MDCT-derived annular perimeter measurements showed similar findings (r = 0.984, p ≤ 0.01, CCC = 0.954, and ICCC = 0.95). The 95% LOA for Bland–Altman analysis were − 8.02 mm and 1.56 mm, with a bias of  − 3.2 mm (Fig. 3A, B).

The area-derived and perimeter-derived annular diameter measurements by 4D-TEE and MDCT also showed similar findings (r = 0.982, p = 0.01, and r = 0.983, p = 0.001, respectively). The area-derived diameter had 95% LOA between − 2.29 and 0.67 mm with a bias of − 0.81 mm, and the perimeter-derived diameter had 95% LOA between − 2.55 and 0.49 mm with a bias of − 1.02 mm (Fig. 3).

As for RCA and LCA ostial height measurements, the 4D TEE and MDCT had a moderate positive linear correlation (r = 0.68, p = 0.01, and r = 0.841, p = 0.01, respectively): the 95% LOA was between − 3.25 and 4.15 mm (with a bias of 0.448 mm) for the former and between − 2.7 and 2.59 mm (with a bias of − 0.05 mm) for the latter (Fig. 3C, D).

**Fig. 4:**
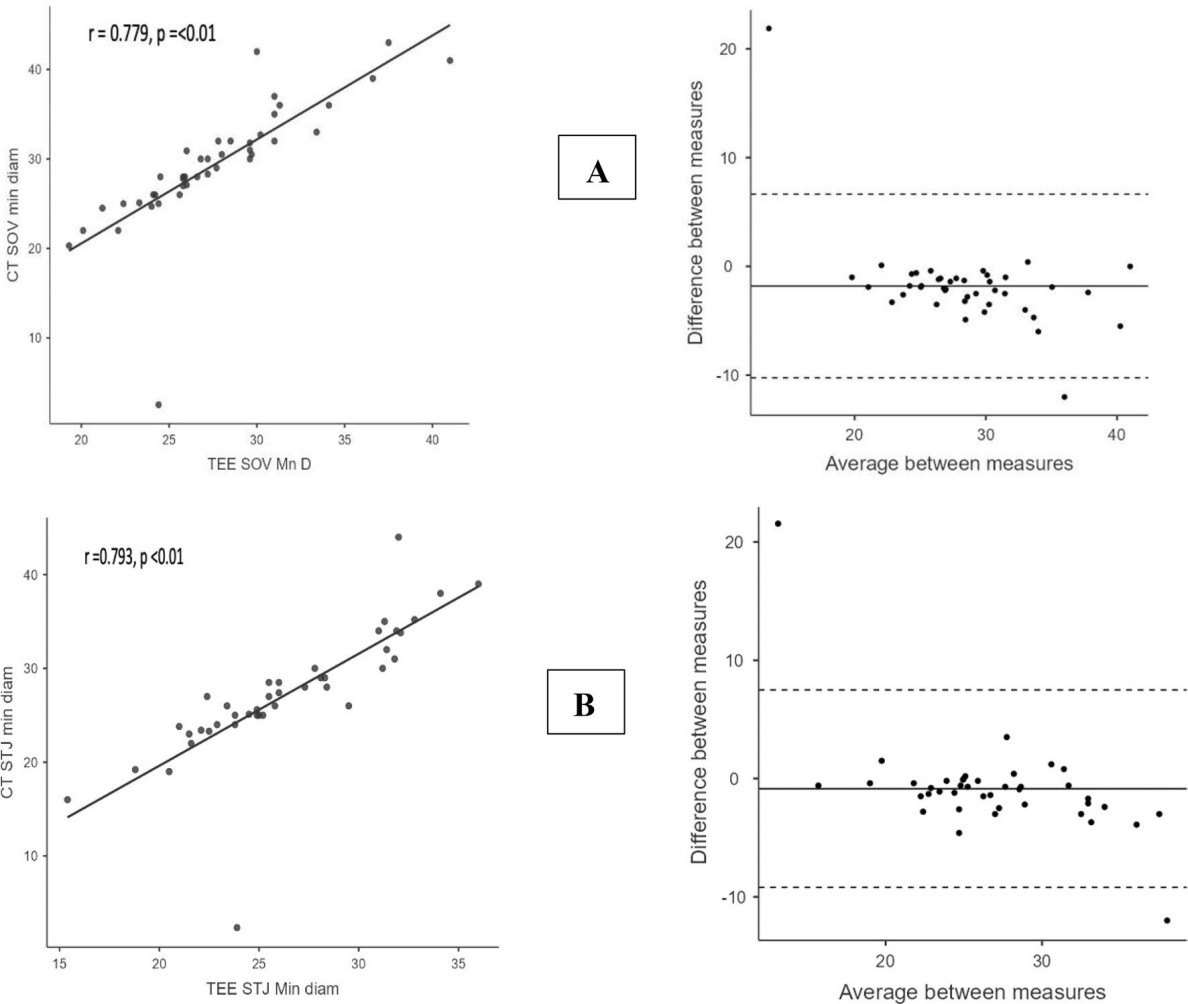
**A** Scatter plot (left) and Bland–Altman plot (right) of SoV minimum diameter and **B** Scatter plot (left) and Bland–Altman plot (right) of STJ minimum diameter. *CT* computed tomography, *SoV* sinuses of  valsalva, *Min* minimum, *Diam* diameter, *TEE* trans-esophageal echocardiography, *STJ* sinotubular junction

For the SoV minimum diameter, there was a statistically significant strong positive linear correlation and moderate agreement in terms of SoV minimum diameter (r = 0.779, p ≤ 0.01, CCC = 0.687, and ICCC = 0.692). Bland–Altman analysis showed 95% LOA between − 10.2 and 6.64 mm, with a bias of − 1.8 mm. Similarly, there was a significantly strong positive linear correlation and good agreement in terms of STJ minimum diameter (r = 0.793, p < 0.01, CCC = 0.723, and ICCC = 0.728). The 95% LOA for the Bland–Altman analysis was between − 9.2 and 7.49 mm, with a bias of − 0.86 mm (Fig. 4A, B).

## Discussion

In this study, we discovered very strong correlations with good agreement between 4D TEE and MDCT-derived annular dimensions: annular area, annular perimeter, and area-derived diameter. Moreover, there were strong correlations and good agreement for LCA height, minimum STJ diameter, and minimum SoV diameters. However, we only detected moderate correlations and agreement with relatively large differences between the 95% LOA for RCA ostial height, maximum STJ diameter, and maximum SoV diameters.

### Aortic annular dimensions

In this study, there were very strong correlations and agreement between 4D TEE and MDCT in the measured annular areas, perimeters, and their respective derived annular diameters. In general, the 4D TEE-derived annular dimensions were smaller than MDCT-derived diameters—it underestimated the annular area by 28.6 mm^2^ (5.7%), the annular perimeter by 3.2 mm (4%), the area-derived annular diameter by 0.8 mm (3.2%), and the perimeter-derived annular diameter by 1.0 mm (3.5%).

There were several comparisons between 4D TEE and MDCT in evaluating aortic root dimensions. Granata et al. was a prospective study that used the same 4D TEE software but on a smaller number of severe symptomatic AS patients (26 patients). They found a statistically significant strong correlation between the two methods in annular areas (r = 0.89, p < 0.0001), annular perimeters (r = 0.89, p < 0.0001), maximum annular diameters (r = 0.79, p < 0.001) and minimum annular diameters (r = 0.81, p < 0.001). The 4D TEE also underestimated MDCT measurements of the annular area by 65.3 mm^2^ (13.6%), annular perimeter by 4 mm (5.2%), maximum annular diameter by 1.2 mm (4.5%), and minimum annular diameter by 2.6 mm (11.3%) [10].

Four previous studies used older software versions. Calleja et al. was a study on 20 normal participants. It showed no significant difference between the two modalities in coronal, sagittal, and average annular dimensions, and the Bland–Altman plot showed good visual agreement [11]. Another retrospective analysis of 47 severe AS patients found significant correlations and good agreement in the area-derived annular diameters (r = 0.88, p < 0.001 and 95% LOA − 4.24 and 1.71 mm) and perimeter-derived annular diameters (r = 0.9, p < 0.001 and 95% LOA between − 3.94 and 1.73 mm) [4]. Similarly, using the same software, Kato et al. retrospectively analyzed the data of 43 severe AS patients undergoing TAVI. They demonstrated strong correlation and good agreement with narrow differences in the annular area (difference = − 6 mm^2^ (1.7%), r = 0.94, p < 0.001, 95% LOA between − 48.3 and 60.2 mm^2^) and the annular perimeter (difference = + 0.1 mm, r = 0.90, p < 0.001, 95% LOA between − 6.5 and 6.3 mm) [12]. Choi et al. found that the 4D TEE underestimated the annular area but with a significant correlation MDCT (difference = 34 mm^2^ (12%), r = 0.98, p = 0.018) [13].

Prihadi et al. used a different software to compare the two modalities in 150 patients with severe AS: the 4D TEE underestimated MDCT-annular dimensions, but both had strong correlation and good agreement in the annular area (difference = − 10.1 mm^2^ (2.2%), r = 0.91, p < 0.001, 95% LOA between − 78.5 and 58.4 mm^2^) and annular perimeter (difference = − 0.3 mm (0.4%), r = 0.83, p < 0.001, 95% LOA between − 8.5 and 8.2 mm) [7]. Khalique et al. reported similar findings with manual analysis of the 4D TEE datasets using a different machine: annular area difference = − 7.9 mm^2^ (2%) (r = 0.94, p < 0.001, 95% LOA between − 65 and 49.1 mm^2^), area-derived annular diameter difference = − 0.22 mm (1%), r = 0.94, p < 0.01) and annular perimeter difference = − 1 mm (1.3%), r = 0.93, p < 0.001, 95% LOA between − 6.0 and 4.0) [14].

### Coronary ostial heights, STJ, and SoV dimensions

In our study, we found good correlations and good agreements with a relatively small difference for the LCA ostial height (r = 0.84, p < 0.01, CCC = 0.83, 95% LOA between − 2.29 and 0.67 mm), minimum STJ diameter (r = 0.793, p < 0.01, CCC = 0.723, 95% LOA between − 9.2 and 7.49 mm) and minimum SoV diameters (r = 0.779, p < 0.01, CCC = 0.687, 95% LOA between − 10.2 and 6.6 mm). But the correlations and agreement were moderate with relatively large differences for RCA ostial height (r = 0.68, p < 0.01, CCC = 0.67, 95% LOA between − 3.25 and 4.15 mm), maximum STJ diameter (r = 0.658, p < 0.01, CCC = 0.594, 95% LOA between − 12.02 and 12 mm) and maximum SoV diameters (r = 0.667, p < 0.01, CCC = 0.537, 95% LOA between − 14.7 and 14.5 mm). We could not come up with a plausible explanation for the moderate correlation in the RCA height versus the good correlation in LCA height, and none of the previous studies reported results on this point: they only published data on the LCA or no data on coronary ostial heights at all.

Fewer studies investigated the correlation between 4D TEE-derived and MDCT-derived coronary arteries’ ostial heights, SoV, and STJ dimensions.

Granata et al. also found a statistically significant moderate positive correlation for RCA ostial height (r = 0.53, p = 0.007) but a weak non-significant correlation for LCA ostial height (r = 0.33, p = 0.1) [10]. Tamborini et al. also reported a strong correlation (r = 0.83, p = 0.01), and a good agreement was good in the LCA height, and the difference was small and non-significant (0.4 mm) [15].

In Prihadi et al. study, 4D TEE underestimated the mean STJ diameter (− 1.4 mm) with good correlation and agreement (r = 0.73, p < 0.001, 95% LOA between − 4.8 and 4.0 mm), and the mean SoV diameter (− 0.7 mm) with good correlation and agreement (r = 0.87, p < 0.001, 95% LOA between − 4.2 and 2.8 mm) [7]. Similarly, Choi et al. study showed that 4D TEE underestimated both maximum STJ diameter (2.69 ± 0.26 vs. 3.19 ± 0.21 cm, r = 0.775, p = 0.042) and maximum SoV diameter (3.16 ± 0.32 vs. 3.92 ± 0.46 cm, r = 0.993, p = 0.007) with significant good correlation [13].

In contrast, Calleja et al. revealed a significant difference in coronary ostial heights measured by 4D TEE and MDCT. The 4D TEE measurements were smaller for the left (11.3 vs. 12.9 mm, p = 0.03) and right (11.6 vs. 13.1 mm, p = 0.001) coronary ostial heights. The mean 4D TEE-derived STJ and SoV diameters were also smaller by 1.4 mm (p < 0.01) and 2.8 mm (p < 0.01), respectively. However, these findings should be interpreted cautiously because the 4D TEE population differed from the MDCT population in this study [11].

Automated analysis of both 4D TEE and CT datasets and their validation is currently an area of active interest in medical research: due to the increased availability of transcatheter structural interventions, especially TAVR, and the increased complexity of aortic root surgeries. Currently, MDCT evaluation of the aortic root is the gold standard despite the drawbacks of ionizing radiation and the use of iodinated contrast, which can be troublesome in patients with renal impairment [1]. The improvements in hardware and software, especially artificial intelligence, made 4D TEE a theoretically viable alternative to MDCT.

To our knowledge, this study is the first to compare 4D TEE and MDCT-derived aortic root dimensions in various aortic root and valve pathologies, including normal root and valve, severe AS, and dilated aortic root. To date, only a few studies have compared semi-automated 4D TEE with MDCT in evaluating aortic root dimensions—they all focused on aortic annular dimensions and were only done in patients with severe AS in preparation for TAVR procedures.

### Study limitations

This study has some limitations. First, it is a single-center study, and its results should be interpreted with caution and further validated in larger multicenter trials. Also, this study did not measure clinical outcomes based on decisions using data obtained from 4D TEE or MDCT. This question can be investigated further through clinical outcome studies. Finally, the limited ability of TEE to detect and quantify calcification can impede assessment in severely calcific aortic roots and valves.

## Conclusion

4D TEE correlates well with MDCT in measuring aortic annular dimensions, coronary ostial height, SoV minor diameter, and sinotubular junction minor diameter. It could replace MDCT if the latter is unavailable or contraindicated. Whether or not this can affect clinical outcomes is yet to be determined.
